# Pyrexia of unknown origin (PUO) and the cost of care in a tertiary care institute in Sri Lanka

**DOI:** 10.1186/s12913-023-09169-1

**Published:** 2023-02-21

**Authors:** Rusiru Premathilaka, Thamal Darshana, Chanil Ekanayake, Kossinnage Chethana Chathurangani, Iroshan Mendis, Sajeethan Perinparajah, Madhushini Shashiprabha, Sachith Nishshanka, Yasoma Tilakaratna, Anuja Premawardhena

**Affiliations:** 1 University Medical Unit, Colombo North Teaching Hospital, Ragama, Sri Lanka; 2 Department of Medical Laboratory Sciences, Faculty of Allied Health Sciences, University of Sri Jayewardenepura, Sri Jayewardenepura, Sri Lanka; 3 Department of Clinical Sciences, Faculty of Medicine, General Sir John Kotelawala Defence University, Dehiwala-Mount Lavinia, Sri Lanka; 4 Department of Medicine, Faculty of Medicine, University of Kelaniya, Kelaniya, Sri Lanka

**Keywords:** Fever, Pyrexia of unknown origin, Direct costs of care

## Abstract

**Background:**

Despite advancements in diagnostic technology, pyrexia of unknown origin (PUO) remains a clinical concern. Insufficient information is available regarding the cost of care for the management of PUO in the South Asian Region.

**Methods:**

We retrospectively analyzed data of patients with PUO from a tertiary care hospital in Sri Lanka to determine the clinical course of PUO and the burden of the cost incurred in the treatment of PUO patients. Non-parametric tests were used for statistical calculations.

**Results:**

A total of 100 patients with PUO were selected for the present study. The majority were males (n = 55; 55.0%). The mean ages of male and female patients were 49.65 (SD: 15.55) and 46.87 (SD: 16.19) years, respectively. In the majority, a final diagnosis had been made (n = 65; 65%). The mean number of days of hospital stay was 15.16 (SD; 7.81). The mean of the total number of fever days among PUO patients was 44.47 (SD: 37.66). Out of 65 patients whose aetiology was determined, the majority were diagnosed with an infection (n = 47; 72.31%) followed by non-infectious inflammatory disease (n = 13; 20.0%) and malignancies (n = 5; 7.7%). Extrapulmonary tuberculosis was the most common infection detected (n = 15; 31.9%). Antibiotics had been prescribed for the majority of the PUO patients (n = 90; 90%). The mean direct cost of care per PUO patient was USD 467.79 (SD: 202.81). The mean costs of medications & equipment and, investigations per PUO patient were USD 45.33 (SD: 40.13) and USD 230.26 (SD: 114.68) respectively. The cost of investigations made up 49.31% of the direct cost of care per patient.

**Conclusion:**

Infections, mainly extrapulmonary tuberculosis was the most common cause of PUO while a third of patients remained undiagnosed despite a lengthy hospital stay. PUO leads to high antibiotic usage, indicating the need for proper guidelines for the management of PUO patients in Sri Lanka. The mean direct cost of care per PUO patient was USD 467.79. The cost of investigations contributed mostly to the direct cost of care for the management of PUO patients.

**Supplementary Information:**

The online version contains supplementary material available at 10.1186/s12913-023-09169-1.

## Background

The syndrome of pyrexia of unknown origin (PUO) comprises a diverse group of medical conditions that share the common feature of incessant fever regardless of basic medical interventions [1]. Many causes of PUO have been described, including infections, non-infectious inflammatory diseases (NIID), malignancies, and miscellaneous [2]. Literature suggests that the relative prominence of each category changes in different geographical and income settings [3]. Malignancies and NIID are the leading contributors to PUO in developed countries [3]. In contrast, infections remain the primary cause of PUO in developing countries. Tuberculosis has been reported as the commonest infection leading to PUO in economically challenging locales [4–7]. Incidentally, a substantial number of PUO patients remain undiagnosed despite modern advances in diagnostic technologies. The underlying aetiology determines the course of PUO. However, the majority of cases with incomprehensible PUO eventually manifest with the sudden cessation of fever [2].

A strenuous analytical approach demanding nonspecific imaging studies and many investigations are required before the PUO work-up, which can be ineffective or misleading; due to the vast aetiology. Failures in diagnosis occur as a result of the lack of proper criteria in investigating cases with PUO [6]. A patient with PUO who has been admitted to a hospital often stays for a prolonged period thus increasing the health care cost. A cost-effective, personalized approach is critical in assesing PUO patients since, without an astute investigation, inappropriate tests might be performed. Many advanced diagnostic technologies such as magnetic resonance imaging, computer tomography, proton-emission tomography, sonography, and numerous serological tests hold a diagnostic value in PUO [8–12]. Blind usage of these techniques with uncertain background information puts an extra burden on patients with prolonged hospitalization. Moreover, it increases the cost of health care, highlighting the importance of proper and validated guidelines for the evaluation and management of PUO. In-depth cost analyses of PUO management are hardly discussed in the literature. In the few studies that have been conducted, PUO management costs have been found to be relatively higher. In a study conducted in Spain, the total real cost of PUO management per patient, including hospitalization, outpatient consultations, and investigations, was 11,167 euros [10]. In Washington, the cost of PUO management per patient has been reported to be much higher (USD 40,295 per patient) among the paediatric group [11].

There is a deficit in research all over the world on this topic, particularly in Sri Lanka. Add to that insufficient data are available regarding the cost of care of PUO in the South Asian region. Therefore, we conducted a retrospective analysis of patients with PUO from a tertiary care institute in Sri Lanka to describe the nature of PUO and the burden of the costs incurred in the management of PUO patients.

## Methods

We conducted a retrospective analysis of the records of patients who had been diagnosed with classic PUO between January 2015 and January 2020 at Colombo-North Teaching Hospital (CNTH), Ragama, the largest hospital in the Gampaha district with a bed strength of 1500. PUO was defined as prolonged unexplained fever lasting more than 3 weeks with axillary temperature exceeding 38.3 °C on several occasions without a diagnosis despite three days of inpatient investigation or three outpatient visits [13]. Patients aged 18 years and above were included in the present study. Children, less than 18 years of age, patients with neutropaenia, nosocomial PUO, HIV/AIDS, and on long-term immunosuppressive therapy were excluded from the study.

### Demographic & clinical data collection

Data were extracted from the hospital records archive including the details of subsequent admissions using a data extraction sheet and investigation summary chart. Demographic data included age, gender, and type of occupation. Moreover, details of the patient’s clinical history with co-morbidities, duration of the fever, the pattern of fever, grade of fever, usage of medication, usage of supportive devices & special therapeutic procedures, details of investigations, duration of hospital stay, and final diagnosis were collected. Grades of fever by rectal temperature were defined as described by Ogoina (2011): mild/low-grade fever (38.1–39.0 ^0^ C), moderate-grade fever (39.1–40.0 ^0^ C), high-grade fever (40.1–41.1 ^0^ C), hyperpyrexia (> 41.1 ^0^ C) [14]. Corresponding rectal temperature was determined by always adding 1 ^0^ C to the axillary temperature as described by Shann and Mackenzie [15].

### Direct cost of care calculation

A micro-costing approach, as practised by Ekanayake et al. was adopted to calculate the direct cost of care for patient management [16]. The main cost categories of the direct cost of care were expenses for medications & other medical items, equipment costs, investigation costs, cost of utilities, and labour costs.

#### Medications and equipment cost

The cost of drugs, other medical items, and equipment was calculated based on the details acquired from the medical supplies division of the Ministry of Health, Sri Lanka [17].

#### Investigation cost

The cost of the investigations included; the cost of laboratory investigations, imaging, and other medical procedures. Investigation costs were calculated using the rates obtained by the Ministry of Health, Sri Lanka [18]. The cost for a particular investigation included the equipment cost, reagent cost, and labour cost.

#### Utility cost

Costs of utilities were calculated per patient per day for space, electricity, water, telephone, security, administrative, and cleaning services. Space rental value was obtained from the National Housing Authority. The costs of other utilities were computed using the corresponding monthly bills divided by the average midnight total of patients to derive the cost for a particular utility per patient per day.

#### Labour cost

Labour cost per minute for each category of staff was calculated by adopting a time-driven, activity-based costing method. All the costs are reported in USD. Average exchange rates for USD to LKR in 2015–2020 were obtained from Central Bank Sri Lanka (SLR/USD: 2015–135.94; 2016 − 145.60; 2017–152.46; 2018–162.54; 2019–178.78; 2020–185.52) [19].

Statistical analyses were performed by Statistical Package for Social Sciences (SPSS) version 26.0. Non-parametric tests were used for statistical calculations. Ethical approval for the study was obtained from the Ethics Review Committee of the Faculty of Medicine, University of Kelaniya, Sri Lanka (P/42/07/2020).

## Results

154 cases of PUO had been recorded during the period of data collection from which only 100 patients fulfilled the inclusion criteria and were recruited for the present study. There were 55 males (55.0%). The ages of the patients ranged from 18 to 77 years with mean ages falling at 49.65 (SD: 15.55) and 46.87 (SD: 16.19) years for male and female patients respectively (Only 99 out of 100 participants had age information available). The majority of the participants were unemployed (76.0%; n = 76). Out of the 100 PUO patients, a final diagnosis has been made in 65 patients (65.0%). Thirty-five patients (35.0%) remained undiagnosed despite extensive investigations. Hospital stays varied widely between 7 and 44 days (mean 15.16, SD: 7.81 days). Interestingly, the mean hospital stay of undiagnosed males was significantly shorter than the diagnosed male patients (p = 0.024). Basic demographic characteristics and details of hospital stays are described in Table 1.

**Table 1:** Descriptive characteristics of PUO patients based on the diagnosis

	Diagnosed	Undiagnosed	p Value^†^
Gender (n)			
Male	38	17	0.343
Female	27	18	
Mean Age (Y)/SD	48.27 (14.94)	48.60 (18.23)	0.703
Male	50.32 (13.62)	48.18(20.28)	0.695
Female	45.44 (16.42)	49.00 (16.64)	0.485
Mean Hospital Stay per admission (D)/SD	16.28 (8.52)	13.09 (5.83)	0.114
Male	16.80 (8.63)	11.4 (5.06)	0.024*
Female	15.56 (8.48)	14.67 (6.19)	0.687
Mean duration of fever – total (D)/SD	42.43 (36.89)	42.57 (43.56	0.595
Male	41.47 (32.00)	47.12 (52.07)	0.750
Female	43.78 (44.91)	38.28 (34.68)	0.676
Mean duration of fever before admission (D)/SD	30.11 (35.37)	32.91 (45.50)	0.463
Male	28.39 (27.59)	38.82 (54.95)	0.971
Female	32.52 (44.56)	27.33 (35.09)	0.358

^†^ level of significance was taken as 0.05 for the Chi-square test and Mann-Whitney U test.

**Fig. 1:**
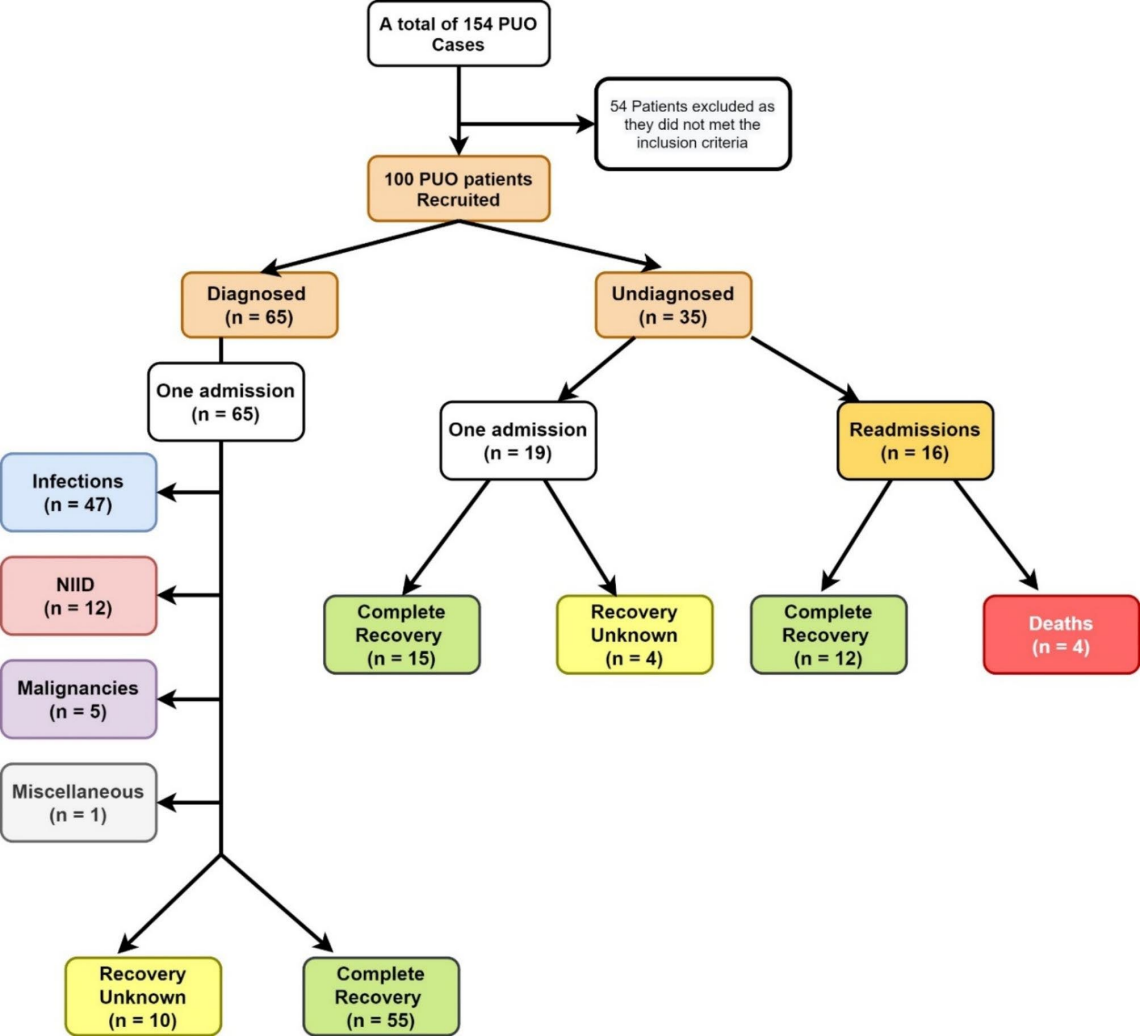
Status of diagnosis and recovery of PUO patients

Duration of fever before hospital admission ranged widely (7-240 days) with a mean of 31.13 (SD: 38.67) days. Likewise, wide variation was also observed in the total duration of fever (21–244 days) which had a mean of 44.47 days (SD: 37.66). Out of 65 patients whose aetiology was identified, the majority were diagnosed with an infection (n = 47; 72.31%) (Fig. 1). Further 12 (18.46%) patients were diagnosed with NIID. Five patients (7.69%) had a malignancy while one (1.54%) was diagnosed with Methotrexate-induced pneumonitis. Extra-pulmonary tuberculosis was the commonest infection (n = 15) diagnosed followed by infective endocarditis (n = 7). Final diagnoses of the detected PUO cases are given in additional file 1. The mean number of days taken to reach a diagnosis after hospital admission was 12.95 (SD: 6.26). In thirty-five patients with PUO, a reason could not be identified. The majority of them (54.3%; n = 19) did not have any co-morbidities at the time of hospital admission. Diabetes mellitus (32.0%; n = 32) was the most common co-morbidity detected among all the PUO patients, followed by hypertension (24.0%; n = 24). Frequencies of different co-morbidities detected among both diagnosed and undiagnosed patients with PUO are given in additional file 2.

Patterns of the fever were also analyzed. Intermittent fever (n = 79; 79.0%) was the commonest pattern followed by ‘a very occasional fever’ (n = 15; 15.0%), relapsing fever (n = 4; 4.0%) and, remittent fever (n = 2; 2.0%). Intermittent fever was significantly more common among diagnosed PUO patients than undiagnosed patients (φ_c_ = 0.291; p < 0.005). The fever pattern of the majority of the patients was persistent during the hospital stay with only four (4.0%) recorded instances of fever pattern change. After admission, 38 (38.0%) patients had “moderate grade” fever, 36 (36.0%) patients had “high grade” fever; 22 (22.0%) had “low grade” fever and one patient had “hyperpyrexia”.

Investigations/procedures resulting in the final diagnosis of PUO patients were also analyzed. Contrast-enhanced computed tomography (CECT) of chest/abdomen/pelvis (n = 15) was found to be the commonest investigation followed by blood culture (n = 12) which lead to the final diagnosis. A complete list of investigations that lead to the final diagnosis is given in additional file 3 with frequencies.

Antibiotics had been prescribed for 90 out of 100 patients after admission. Further, antacids, analgesics, anti-tuberculosis therapy, steroids, non-steroidal anti-inflammatory drugs, immunosuppressants, and deep vein thrombosis– prophylactic drugs have also been prescribed for 73, 69, 10, 10, 5, 4, and 2 patients respectively. Ceftriaxone (n = 47) was the commonest first-line antibiotic prescribed, followed by co-amoxiclav (n = 11) and meropenem (n = 9). Strong significant positive correlations (p < 0.001) were observed between the total hospital stay of the PUO patients and duration of fever after admission; total duration of antibiotic usage; duration of antibiotic usage after admission; days taken to final diagnosis respectively. Ninety-eight PUO patients (98%) were discharged from the hospital. Two patients (2%) left the hospital against medical advice. Sixteen of the 35 undiagnosed patients (45.7%) had been readmitted to the hospital. Upon later inquiry over the phone, complete recovery was noted among 82 patients (55 diagnosed; 27 undiagnosed patients). Four deaths were also noted in the undiagnosed category. The cause of death in all four cases was sepsis. Nevertheless, we were unable to verify the status of recovery in 14 patients (10-diagnosed; 4 undiagnosed patients) (14%) due to the unresponsiveness and unavailability of contact details (Fig.  1).

Details of expenses of management of PUO were available for only 64 patients in the present study. The mean direct cost of care per PUO patient during the study period (2015–2020) was USD 467.79 (SD: USD 202.81). The mean direct cost of care per PUO patient per day was USD 28.84 (SD: USD 9.91) whereas the cost of care including only the cost of labour and utilities per PUO patient per day was USD 11.46 (SD: USD 2.86). Even though the mean direct cost of care in diagnosed PUO patients (USD 492.91; SD: 202.83) was higher than that of undiagnosed PUO patients (USD 435.49; SD: 201.79), the Mann-Whitney U test was unable to demonstrate any significant difference between groups (p =0.279). Of the different categories, the cost of investigations (Laboratory, imaging, and clinical) contributed mostly to the direct cost of care for the management of PUO patients (Table 2).

**Table 2:** Different categories of expenses during the study period (2015–2020) for the management of PUO patients

Category of expenses	Mean cost per PUO patient (USD) (SD)	Percentage contribution per patient
Cost of medications and equipment	45.33 (40.13)	8.03%
Cost of investigations (Lab and clinical)	230.26 (114.68)	49.31%
Cost of labour	45.68 (22.32)	9.94%
Cost of utilities (Water, electricity, telephone, security, administration, cleaning, and space)	147.23 (72.89)	31.99%
Mean direct cost of care per patient (2015–2020)	467.79 (202.81)	

The mean direct cost of care was higher among patients who had been diagnosed with an infection (USD 497.15) than those with NIID, malignancies, and who had not been diagnosed. However, the Kruskal-Wallis H test was unable to find any significant difference in the mean ranks of the direct cost of care between the etiological categories of PUO in the present study (Table 3). Furthermore, a significant positive correlation (Spearman’s ρ = 0.607; p < 0.001) was observed between the hospital stay and the direct cost of care of PUO management.

**Table 3:** Direct cost of care of PUO across different etiological categories and undiagnosed cases

	Infection (n = 25)	NIID (n = 8)	Malignancy (n = 3)	Undiagnosed (n = 28)	p-Value*
Mean cost of care for medications and equipment in USD (SD)	48.88 (39.50)	34.47 (41.11)	47.97 (43.62)	44.96 (41.83)	0.721
Mean cost of care for investigations (lab and clinical) in USD (SD)	234.84 (117.11)	259.88 (141.33)	224.41 (29.23)	218.33 (113.24)	0.875
Mean cost of care for labour in USD (SD)	50.49 (26.90)	47.69 (21.87)	43.81 (18.52)	41.01 (17.99)	0.671
Mean cost of care for utilities (Water, electricity, telephone, security, administration, cleaning, and space) in USD (SD)	162.94 (88.02)	151.12 (68.67)	140.78 (60.01)	132.79 (59.72)	0.673
Mean direct cost of care in USD (SD)	497.15 (223.73)	493.15 (166.08)	456.98 (143.50)	435.49 (201.80)	0.735

*p < 0.05 of the Kruskal-Wallis H test was taken as significant

## Discussion

Pyrexia of Unknown origin continues to be a diagnostic and management challenge for clinicians around the globe. Diverse aetiology further complicates the diagnosis of PUO. Clinicians must have an idea of the probable diagnosis even if the exact aetiology may not be known for a long time. Most of the diagnosed PUO cases in the present study were caused by infectious diseases (n = 47; 72.3%) with tuberculosis being the commonest infection (n = 15; 31.91%). Given its commonness, it is no surprise that tuberculosis accounted for many PUO cases in Sri Lanka. Similar findings have been reported from Kolkata, Chandigarh (India), Karachi (Pakistan), Ahvaz (Iran), and Dhaka (Bangladesh) in which 41.6%, 45.3%, 65.2%, 70%, 31.9%, and 25.0% of infectious causes of PUO were due to tuberculosis respectively [4–7, 20, 21]. In the present study, the diagnosis of tuberculosis had been made with evidence of chest X-ray, biopsy examination, tuberculosis culture, and nucleic acid amplification (X Pert-MTB/RIF) [22]. The average time taken to reach a final diagnosis in PUO varies widely in different reports. In the present study, it was 12.95 days. Shorter time durations than the present study have been reported in Kolkata (5.64 days; SD: 3.2 days) and Dhaka (8.2 days) [6, 20]. Incidentally, the average time duration taken to reach a diagnosis of PUO was 19 days (SD: 14 days) and 53.54 days (SD: 152.24 days) in Karachi and Saudi Arabia [4, 23].

More than one-third of the PUO patients in the present study (n = 35;35%) remained undiagnosed during hospitalization which is higher than that (0-27.4%) reported from previous South Asian studies [4, 24]. Interestingly, the majority (54.3%; n = 19) of the undiagnosed PUO patients in the present study did not show any underlying pathologies apart from fever during hospital admission. Fever in the undiagnosed patients in the present study had ceased while under investigation prompting the early discharge before arriving at a diagnosis. It has led to a shorter mean hospital stay in the undiagnosed group than that of the diagnosed group in the present study. The proportion of PUO patients who remain undiagnosed has increased in the Western hemisphere despite increasing advances in technology [25]. However, recent evidence suggests that the prognosis of these undiagnosed PUO patients is likely to be good despite the continuance of symptoms [26]. The proportions of PUO cases caused by NIID and malignancies in the present study were 12% (n = 12) and 5% (n = 5) respectively. South Asian reports have shown comparatively higher proportions of PUO caused by NIID (11.0–20.1%) and malignancies (9.0–21.5%) than in the present study [4, 7, 24]. Interestingly, the present study identified only three (4.6%) cases of PUO caused by connective tissue disorders. Similar findings have been observed in Dhaka and northern India, where the contributions of connective tissue disorders to the aetiology of PUO were 3.0% (n = 1) and 3.9% (n = 6) [7, 20]. However, the contributions of connective tissue disorders to the aetiology of PUO were higher in studies done in Saudi Arabia (14.3%; n = 14) and Karachi (12.7%; n = 27) [4, 23].

The contribution of imaging techniques to diagnose the causes of PUO was higher than any other investigation in the present study with CECT chest/abdomen/pelvis (n = 15; 23.1%) being the commonest investigation that led to a diagnosis. Similar observations have also been reported in studies from Saudi Arabia and India [23, 27]. Recent developments in imaging techniques have permitted the early detection of multiple diseases and conditions which were previously hard to diagnose. Usage and availability of positron emission tomography-computed tomography (PET-CT) has increased as of late and is often used to detect the metabolically active center of inflammation, infection, or malignancy, as likely causes of PUO [24]. However, the limited availability and heterogeneous accessibility of PET-CT in an economically challenging set-up like Sri Lanka has hindered the utility of PET-CT in diagnosing PUO.

Supportive management is recommended for PUO until the cause has been determined. Regimens of steroids and antibiotics are not advised as they can mask the signs and symptoms of the underlying disease condition. Empirical therapy in PUO is advised as appropriate only in three instances: antimicrobials for patients with signs of sepsis and infective endocarditis, regimens of steroids for suspected temporal arteritis, and anti-tuberculous therapy for suspected miliary tuberculosis or central nervous system tuberculosis [26]. Even though, bacterial infections were identified only in 47% (n = 47) of PUO patients, antibiotics had been prescribed for 90% (n = 90) patients in the present study. We were unable to ascertain the rationale for the excess use of antibiotics from the available records. The outcome of the PUO and the status of recovery could not be ascertained in fourteen patients (14%) in the present study due to the unavailability of records and unresponsiveness of patients highlighting the dire need for a thorough documentation system.

The present study explored only the direct cost of care for PUO patients. Indirect costs such as out-of-pocket payments, productivity losses, cost of the career, and travel costs have not been considered when computing the cost of care of PUO which we believe is a limitation of the study. The mean direct cost of care per PUO patient in the present study was USD 467.79 with no significant difference between diagnosed (USD 492.91) and undiagnosed PUO patients (USD 435.49). The mean direct cost of care per PUO patient in the present study (USD 467.79) was almost thrice the per capita health expenditure of Sri Lanka (USD 157.47) in 2018 [28]. Also, the direct cost of care per PUO patient per day in the present study (USD 28.84) was higher than the cost per inpatient bed day in World Health Organization (WHO) – CHOICE model (USD 23.31) for Sri Lanka [29]. Incidentally, WHO – CHOICE model for inpatient care was developed in 2007 and based on the type of hospital, ownership, bed occupancy rate, the total number of inpatient admissions, and, the average length of stay. However, in the present study, the direct cost of care was estimated based on the micro-costing of expenses for medications & other medical items, equipment, investigations, utility, and labour. Hospital stay is a major determinant of the direct cost of care for PUO management. In the present study, we observed a significant positive correlation between the hospital stay and the direct cost of care for PUO management (Spearman’s ρ = 0.607; p < 0.001). It highlights the fact that the implementation of an effective management strategy and reducing hospital stays could potentially reduce the direct cost of care for PUO management.

There is a dearth of information on the cost of care in PUO globally. A much higher direct cost of care has been reported in a study conducted by a Spanish group in which the average direct cost of care was EUR 11,016 (USD 12,997) [10]. The large difference in “direct cost of care” between the present and the Spanish studies could be due to the higher utility costs in Western countries than in Sri Lanka. Incidentally, in the present study, 49.31% of the average direct cost of care was determined by the cost of investigations whereas it was only 12.7% in the Spanish study [10]. The paucity of information on the cost of care of PUO in the South Asian region precluded us from effectively analyzing the findings of the present study concerning the regional data. The inclusion of fluorodeoxyglucose (FDG)-PET/CT in the diagnostic workup of PUO has been studied and if conducted early found to be effective in reducing the cost of care on several occasions. However, the present study was unable to examine this hypothesis due to the inaccessibility and unavailability of (FDG)-PET/CT.

## Conclusion

Infections accounted for most of the cases of PUO with extrapulmonary tuberculosis leading the list. PUO leads to high antibiotic usage, stressing the need for evidence-based guidelines including a meticulous diagnostic approach for the management of PUO patients in Sri Lanka. The cost of investigations contributed mostly to the direct cost of care for the management of PUO patients.

## Electronic supplementary material

Below is the link to the electronic supplementary material.

Supplementary Material 1

Supplementary Material 2

Supplementary Material 3

## Data Availability

The datasets generated during and analyzed during the current study are not publicly available due to them containing information that could compromise research participant privacy/consent, but aggregated data are available from the corresponding author on reasonable request.

## Abbreviations

PUO: Pyrexia of unknown origin
NIID: Non-infectious inflammatory disease
WHO: World Health Organization
CNTH: Colombo-North Teaching Hospital
CECT: Contrast-enhanced computer tomography
PET-CT: positron emission tomography-computed tomography
FDG: fluorodeoxyglucose
USD: United States Dollar
EUR: Euro
SD: Standard deviation

## References

[1] Beresford RW, Gosbell IB (2016). Pyrexia of unknown origin: causes, investigation and management. Intern Med J.

[2] Mulders-Manders C, Simon A, Bleeker-Rovers C (2015). Fever of unknown origin. Clin Med (Lond).

[3] Unger M, Karanikas G, Kerschbaumer A, Winkler S, Aletaha D (2016). Fever of unknown origin (FUO) revised. Wien Klin Wochenschr.

[4] Mahmood K, Akhtar T, Naeem M, Talib A, Haider I, Siraj Us S (2013). Fever of unknown origin at a teritiary care teaching hospital in Pakistan. Southeast Asian J Trop Med Public Health.

[5] Alavi SM, Nadimi M, Zamani GA (2013). Changing pattern of infectious etiology of fever of unknown origin (FUO) in adult patients in Ahvaz, Iran. Casp J Intern Med.

[6] Bandyopadhyay D, Bandyopadhyay R, Paul R, Roy D (2011). Etiological study of fever of unknown origin in patients admitted to medicine ward of a teaching hospital of eastern India. J Glob Infect Dis.

[7] Pannu AK, Golla R, Kumari S, Suri V, Gupta P, Kumar R (2021). Aetiology of pyrexia of unknown origin in north India. Trop Doct.

[8] Wolf H, Graninger W (2003). Cost-effectiveness in diagnosis of patients with long-standing fever. Wien Med Wochenschr.

[9] Bharucha T, Rutherford A, Skeoch S, Alavi A, Brown M, Galloway J (2017). Diagnostic yield of FDG-PET/CT in fever of unknown origin: a systematic review, meta-analysis, and Delphi exercise. Clin Radiol.

[10] Becerra Nakayo EM, García Vicente AM, Soriano Castrejón AM, Mendoza Narváez JA, Talavera Rubio MP, Poblete García VM (2012). Analysis of cost-effectiveness in the diagnosis of fever of unknown origin and the role of 18F-FDG PET–CT: a proposal of diagnostic algorithm. Rev Esp Med Nucl Imagen Mol (English Edition).

[11] Chen J, Wang Q (2019). Cost-effectiveness analysis of 18F-FDG PET/CT in the diagnosis of fever of unknown origin in China. J Nucl Med.

[12] Szymanski AM, Clifford H, Ronis T (2020). Fever of unknown origin: a retrospective review of pediatric patients from an urban, tertiary care center in Washington, DC. World J Pediatr.

[13] Durack DT, Street AC (1991). Fever of unknown origin–reexamined and redefined. Curr Clin Top Infect Dis.

[14] Ogoina D (2011). Fever, fever patterns and diseases called ‘fever’--a review. J Infect Public Health.

[15] Shann F, Mackenzie A (1996). Comparison of rectal, axillary, and forehead temperatures. Arch Pediatr Adolesc Med.

[16] Ekanayake C, Pathmeswaran A, Kularatna S, Herath R, Wijesinghe P (2019). Challenges of costing a Surgical Procedure in a Lower-Middle-Income Country. World J Surg.

[17] Lanka MoHS, PRICE LIST OF MSD ITEMS -. JANUARY 2020. In: Division MS, editor. Colombo: Ministry of Health Sri Lanka; 2020. p. 435.

[18] Lanka MoHS. Revision of charges for the services provided by Hospitals in paying wards and for non-sri lankan Citizens. editor. Colombo: Ministry of Health Sri Lanka; 2017. p. 13. Ministry of Health NIM.

[19] Annual Reports. : Central Bank of Sri Lanka. https://www.cbsl.gov.lk/en/publications/economic-and-financial-reports/annual-reports (2021). Accessed 25 Feb 2021.

[20] Rahim M, Ahmed AKM, Hossain M, Rahman M, Ghosh S, Nazneen S (2016). Aetiology of fever of unknown origin: one-year experience in a Tertiary Care Hospital of Bangladesh. BIRDEM Med J.

[21] Kejariwal D, Sarkar N, Chakraborti SK, Agarwal V, Roy S (2001). Pyrexia of unknown origin: a prospective study of 100 cases. J Postgrad Med.

[22] Lee JY (2015). Diagnosis and treatment of extrapulmonary tuberculosis. Tuberc Respir Dis (Seoul).

[23] Moawad MA, Bassil H, Elsherif M, Ibrahim A, Elnaggar M, Edathodu J (2010). Fever of unknown origin: 98 cases from Saudi Arabia. Ann Saudi Med.

[24] Prabath Kumar D, Arun Kumar D, Rajeshwari K, Neeharika D, Sindhu G, Sreevidya B (2016). Fever of unknown origin (FUO): evolution of case definition, changing aetiological spectrum. JCSR.

[25] Bleeker-Rovers CP, Vos FJ, de Kleijn E, Mudde AH, Dofferhoff TSM, Richter C (2007). A prospective multicenter study on fever of unknown origin: the yield of a structured diagnostic protocol. Med (Baltim).

[26] Fernandez C, Beeching NJ (2018). Pyrexia of unknown origin. Clin Med (Lond).

[27] Rupali P, Garg D, Abraham O, David T, Surekha V (2016). Etiology of Classic Fever of unknown origin among immunocompetent adults from India. Open Forum Infect Dis.

[28] Bank TW, The World Bank Group. Current health expenditure per capita (current US$) - Sri Lanka Washington, D.C., U.S.: ; 2018 [Available from: https://data.worldbank.org/indicator/SH.XPD.CHEX.PC.CD?locations=LK. Accessed 05 Mar 2021.

[29] Stenberg K, Lauer JA, Gkountouras G, Fitzpatrick C, Stanciole A (2018). Econometric estimation of WHO-CHOICE country-specific costs for inpatient and outpatient health service delivery. Cost Eff Resour Alloc.

