# Supplementary material for: Pyrexia of unknown origin (PUO) and the cost of care in a tertiary care institute in Sri Lanka
Source: BMC Health Serv Res. 2023 Feb 21;23:177. doi: 10.1186/s12913-023-09169-1 (PMC9945736; doi:10.1186/s12913-023-09169-1)
Supplement: Supplementary file 1 — Supplementary Material 1 [file 12913_2023_9169_MOESM1_ESM.docx]

**Additional file 1**

**Additional table 1**: Final diagnoses of the PUO patients

| Diagnosis | Number of patients  N = 100 | % of specific diagnostic category |
| --- | --- | --- |
| Infection | **47** |  |
| Extra-pulmonary tuberculosis | 15 | 31.91% |
| Infective endocarditis | 7 | 14.89% |
| Melioidosis | 5 | 10.64% |
| Rickettsial infection | 4 | 8.51% |
| Lung abscess | 4 | 8.51% |
| Atypical pneumonia | 3 | 6.38% |
| Bacterial prostatitis | 3 | 6.38% |
| Pelvic inflammatory disease | 2 | 4.26% |
| Acute pyelonephritis | 1 | 2.13% |
| Typhoid | 1 | 2.13% |
| Chronic sinusitis | 1 | 2.13% |
| Liver abscess | 1 | 2.13% |
|  | | |
| Non-infectious inflammatory conditions | **12** |  |
| Auto-immune thyroiditis | 4 | 33.33% |
| Still’s disease | 2 | 16.66% |
| ANCA vasculitis | 2 | 16.66% |
| Systemic lupus erythematosus | 1 | 8.33% |
| Immune thrombocytopenic purpura | 1 | 8.33% |
| Panniculitis | 1 | 8.33% |
| Haemophagocytic lymphohistiocytosis | 1 | 8.33% |
|  | | |
| Malignancies | **5** |  |
| Non-Hodgkin lymphoma | 3 | 60.0% |
| Multiple myeloma | 1 | 20.0% |
| Hepatocellular carcinoma | 1 | 20.0% |
|  | | |
| Miscellaneous | **1** |  |
| Methotrexate induced pneumonitis | 1 | 100.0% |
|  |  |  |
| Undiagnosed (True PUO) | **35** |  |
