# Supplementary material for: Pyrexia of unknown origin (PUO) and the cost of care in a tertiary care institute in Sri Lanka
Source: BMC Health Serv Res. 2023 Feb 21;23:177. doi: 10.1186/s12913-023-09169-1 (PMC9945736; doi:10.1186/s12913-023-09169-1)
Supplement: Supplementary file 2 — Supplementary Material 2 [file 12913_2023_9169_MOESM2_ESM.docx]

**Additional file 2**

**Additional table 2:** Frequencies of different Co-morbidities presented among PUO patients at the time of Hospital Admission

| **Co-morbidity** | **Diagnosed PUO patients (n = 65)** | **Undiagnosed PUO patients (n=35)** | **Total**  **(n = 100)** |
| --- | --- | --- | --- |
|  | **Frequency**  **(n)/(%)** | **Frequency**  **(n)/(%)** | **Frequency**  **(n)/(%)** |
| Diabetes mellitus | 22 (33.8%) | 10 (28.6%) | 32 (32.0%) |
| Hypertension | 17 (26.2%) | 7 (20.0%) | 24 (24.0%) |
| Dyslipidemia | 5 (7.7%) | 3 (8.6%) | 8 (8.0%) |
| Ischaemic heart disease | 5 (7.7%) | 2 (5.7%) | 7 (7.0%) |
| Bronchial Asthma | 6 (9.2%) | 1 (2.9%) | 7 (7.0%) |
| Autoimmune thyroiditis | 3 (4.6%) | 0 (0.0%) | 3 (3.0%) |
| Chronic kidney disease | 3 (4.6%) | 0 (0.0%) | 3 (3.0%) |
| Hypothyroidism | 2 (3.1%) | 0 (0.0%) | 2 (2.0%) |
| Rheumatoid arthritis | 2 (3.1%) | 0 (0.0%) | 2 (2.0%) |
| Dilated cardiomyopathy | 0 (0.0%) | 1 (2.9%) | 1 (1.0%) |
| Megaloblastic anaemia | 0 (0.0%) | 1 (2.9%) | 1 (1.0%) |
| Osteoarthritis | 0 (0.0%) | 1 (2.9%) | 1 (1.0%) |
| Rheumatoid valvular disease | 0 (0.0%) | 1 (2.9%) | 1 (1.0%) |
| Spondylolisthesis | 0 (0.0%) | 1 (2.9%) | 1 (1.0%) |
| Cerebrovascular disease | 1 (1.5%) | 0 (0.0%) | 1 (1.0%) |
| Chronic liver disease | 1 (1.5%) | 0 (0.0%) | 1 (1.0%) |
| Colorectal carcinoma | 1 (1.5%) | 0 (0.0%) | 1 (1.0%) |
| Myelodysplastic syndrome | 1 (1.5%) | 0 (0.0%) | 1 (1.0%) |
| Prostatic hyperplasia | 1 (1.5%) | 0 (0.0%) | 1 (1.0%) |
| **No Co-morbidity** | 24 (36.9%) | 19 (54.3%) | 43 (43.%) |
