# Supplementary material for: Pyrexia of unknown origin (PUO) and the cost of care in a tertiary care institute in Sri Lanka
Source: BMC Health Serv Res. 2023 Feb 21;23:177. doi: 10.1186/s12913-023-09169-1 (PMC9945736; doi:10.1186/s12913-023-09169-1)
Supplement: Supplementary file 3 — Supplementary Material 3 [file 12913_2023_9169_MOESM3_ESM.docx]

**Additional file 3**

**Additional table 3:** Frequency of different investigations leading to the final diagnosis of PUO

| **Type of the investigation** | **Frequency of each investigation**  **(n)/(%)** |
| --- | --- |
| **Imaging techniques/procedures** |  |
| CECT chest/abdomen/pelvis | 15 (23.1%) |
| 2-D (two-dimensional) echocardiography- Trans-thoracic | 7 (10.8%) |
| Chest X-ray | 7 (10.8%) |
| Trans-oesophageal echocardiogram (TOE) | 6 (9.2%) |
| CECT chest | 5 (7.7%) |
| Kidney, ureter, and bladder X-ray | 5 (7.7%) |
| Ultrasound scan – abdomen | 5 (7.7%) |
| Ultrasound scan – neck | 2 (3.1%) |
| Ultrasound scan – Transvaginal | 2 (3.1%) |
| Ultra sound scan – chest | 1 (1.5%) |
| **Microbiological tests** |  |
| Blood culture | 12 (18.5%) |
| Tuberculosis culture | 8 (12.30%) |
| High vaginal swab culture | 2 (3.1%) |
| **Serological investigations** |  |
| Meliodosis antibody | 5 (7.7%) |
| Rickettsial antibody | 4 (6.2%) |
| Prostate-specific antigen | 3 (4.6%) |
| Mycoplasma antibody | 3 (4.6%) |
| Anti-nuclear antibody | 3 (4.6%) |
| Perinuclear anti-neutrophil cytoplasmic antibodies (P- ANCA) | 2 (3.1%) |
| S.Typhi H antigen | 1 (1.5%) |
| **Hematological investigations** |  |
| Bone marrow (Trephine) biopsy | 7 (10.8%) |
| Blood picture | 2 (3.1%) |
| **Histopathological investigations** |  |
| Skin biopsy | 2 (3.1%) |
| Renal biopsy | 2 (3.1%) |
| Lymph node biopsy | 1 (1.5%) |
| **Molecular investigations** |  |
| X Pert (MTB/RIF) nucleic acid amplification | 7 (10.8%) |
| Epstein–Barr virus PCR | 1 (1.5%) |
